# Dengue in an area of the Colombian Caribbean

**Published:** 2015-03-30

**Authors:** Nelson Alvis-Guzman, Heidi Rodríguez-Barreto, Salim Mattar-Velilla

**Affiliations:** 1 Group of Health Economics, Faculty of Economic Sciences, Universidad de Cartagena Cartagena. Colombia; 2 Institute of Tropical Biological Research, Universidad de Córdoba Montería. Córdoba, Colombia

**Keywords:** Colombia, Dengue Fever, Classical Dengue Fever, Surveillance

## Abstract

**Background:**

In Colombia, dengue is an endemic disease and the four serotypes have been reported.

**Objective:**

To describe the frequency and severity of dengue in an area of the Colombian Caribbean (Department of Cordoba)

**Methods:**

A retrospective study was conducted. Two data sources were analysed: The database from the Direction of Health in Córdoba, and clinical registers of patients diagnosed with haemorrhagic fevers and fevers of unknown origin in reference hospitals.

**Results:**

The mean incidence of dengue between 2003-2010 was 36.5 cases/10^5^ inhabitants (CI95%: 34.3-37.5) and adjusted for sub-reporting, could be between 178.5 and 521.6. The mean incidence of severe dengue was 4.7 cases/10^5^ inhabitants (CI95%: 4.3-5.0). Mean mortality rate due to dengue was 0.3 cases/10^5^ inhabitants. The fatality rate was below 1%. The mean total leukocyte count in patients with dengue was 6,181 mm^3^ (CI95%: 5,973-6,389) and with severe Dengue was 4,729 mm^3^ (CI95%: 4,220-5,238). The average platelet count in patients with Dengue was 118,793/mm^3^ (CI95%: 107,255-130,331) and in patients with Severe Dengue 77,655 (CI95%: 59,640-95,670). Both differences were statistically significant (*p* <0.05). The frequency of laboratories test per patient in patients with Dengue and severe Dengue were different.

**Conclusion:**

The department of Cordoba is a highly endemic zone of Dengue and severe Dengue in the Colombian Caribbean. Moreover, the results show significant differences between dengue and severe dengue so much in tests as in frequency of use of healthcare services.

## Abstract (es)

**Antecedentes:**

en Colombia el Dengue es una enfermedad endémica en la que se han descrito los cuatro serotipos que la producen.

**Objetivo:**

Describir la frecuencia y severidad del Dengue en el departamento de Córdoba de la región caribe colombiana.

**Métodos:**

Se llevó a cabo un estudio retrospectivo a partir del análisis de dos fuentes de datos: a) la base de datos de los casos reportados a la dirección de salud departamental y b) los registros clínicos de pacientes con diagnóstico de fiebres hemorrágicas y fiebre de origen desconocido., en hospitales de referencia del departamento de Córdoba.

**Resultados:**

La incidencia media de dengue entre 2003-2010 fue de 36.5 casos por cien mil habitantes (IC95%: 34.3-37.5) y ajustando por sub-registro podría estar entre 178.5 y 521.6. La incidencia media de dengue grave fue de 4.7 casos por cien mil habitantes (IC95%: 4.3-5.0). La tasa promedio de mortalidad por el dengue durante el período fue de 0.3 casos por cien mil habitantes. El recuento leucocitario total promedio en los pacientes con dengue fue 6,181 x mm^3^ (IC95%: 5,973-6,389) y con dengue grave fue 4,729 x mm^3^ (IC95%: 4,220-5,238). El recuento medio de plaquetas en pacientes con Dengue fue 118.793 / mm^3^ (IC95%: 107,255-130,331) y en pacientes con Dengue Grave fue 77,655 (IC95%: 59,640-95,670). Las diferencias fueron estadísticamente significativas (*p* <0.05). La frecuencia de los laboratorios de pruebas por paciente en los pacientes con dengue y dengue grave fueron diferentes.

**Conclusión:**

El departamento de Córdoba es una zona altamente endémica de dengue y dengue grave en el Caribe colombiano. Los resultados muestran diferencias significativas en algunas pruebas de laboratorio entre el dengue y el dengue severo.

## Introduction

Dengue is a Tropical infectious disease caused by four serotypes of the dengue virus (DENV-1, DENV-2, DENV-3, and DENV-4) 1. It is transmitted through the bite of the *Aedes aegypti* mosquito vector 1. The incidence of dengue has grown dramatically around the world in recent decades. The actual numbers of dengue cases are underreported and many cases are misclassified. One recent estimate indicates 390 million dengue infections per year (95% credible interval 284-528 million), of which 96 million (67-136 million) manifest clinically (with any severity of disease) 2 . The 2009 World Health Organization (WHO) criteria classify dengue according to levels of severity: dengue without warning signs; dengue with warning signs (abdominal pain, persistent vomiting, fluid accumulation, mucosal bleeding, lethargy, liver enlargement, increasing hematocrit with decreasing platelet); and severe dengue (SD) (dengue with severe plasma leakage, severe bleeding, or organ failure) 3.

Because of its high epidemiological and economic impact, dengue occupies the fifth place in the list of unattended tropical diseases in the Americas in terms of disability-adjusted life years (DALY) 4.

According to the PAHO, between 2001 and 2007, close to 30 countries in the Americas reported 4,332,731 cases of dengue; of which 106,037 (2.4%) corresponded to SD, with 1.2% mortality rate. Argentina, Brazil, Chile, Paraguay, and, Uruguay reported 64.6% of the dengue cases. Bolivia, Colombia, Ecuador, Peru, and, Venezuela reported 19.0% (819,466) of the dengue cases with 58% of the cases of SD and 306 deaths 3.

In Colombia, dengue is an endemic disease and the four serotypes have been reported. Between 2004 and 2006, it was the country with the highest number of cases of SD and deaths due to this cause in America 5. According to the Dengue Epidemiological Bulletin, to epidemiological week N°53 of 2014 the following have been reported in the National Health Institute's Public Health Surveillance System (Sistema de Vigilancia en Salud Pública - SIVIGILA): 110,473 total cases of dengue, 107,696 dengue cases (97,5%) and 2,777 (2.5%) SD, and 109 deaths with laboratory confirmation for 3.9% fatality, far above that of 2010 (2.3%) 6. However, the situation can worsen given the current high level of sub-reporting 7. In Córdoba, department in the tropical region of Colombia's Caribbean zone, in 2002 some 2,664 cases of dengue (128 x 100,000) were reported, along with 197 cases of SD (9.5 cases x 100,000 inhabitants). This study describes the frequency and severity of dengue in the department of Córdoba in Colombia's Caribbean (2003-2010) with aim to inform public health policies. 

## Materials and Methods

A descriptive retrospective study was conducted to analyze the epidemiological characteristics of dengue between 2003 and 2010 in an area of the Colombian Caribbean, the department of Córdoba. The study's reference population was that from the Department of Córdoba, Colombia with a land surface of 23,980 km², located in Colombia's northwest on the vast plains of the Caribbean (132,000 km²) at 7° 22' and 9° 26' latitude north and 74° 47' and 76° 30' longitude west of Greenwich, which in 2010 had 1,582,784 inhabitants, distributed in 30 municipalities 8. 

Two data sources were analyzed for the study: a) Database from the Direction of Health in the Department of Córdoba with 4,874 records of confirmed cases of dengue, reported from 2003 to 2010 (Data A). Of these records, only 1,965 (40.3%) had complete data that permitted estimating demographic and epidemiological parameters. To estimate incidence, population data were taken from population projections from Colombia's National Statistics Department (DANE), based on the 1993 census 8. b) Clinical histories of patients diagnosed with hemorrhagic fevers and fevers of unknown origin in the main reference hospitals in the Department of Córdoba (Montería, Planeta Rica, Sahagún, Tierralta, Lorica, and Cereté) between 2003-2007. A total of 2,604 clinical histories were identified of patients with presumptive or confirmed diagnosis of dengue (CIE-10, 210, and 220). The 2,604 histories were assumed as universe of observations and from these, a random sample 230 (9%) were selected (Data B), which had complete information, regarding clinical and epidemiological parameters. From analysis of the clinical histories, we studied socio demographic variables, age, sex, place of residence, and dengue classification (dengue and SD), according to WHO (2009).

To correct the sub-reporting of notification of cases and estimate the actual cases of dengue in the department of Córdoba, we used the adjustment factor proposed by Camacho *et al* 7 *.* The following formula expresses the adjustments of the sub-reporting: Actual number of cases = 9 x cases recorded according to the Córdoba Direction of Health. Information from both sources was entered onto a database by using Microsoft Office Excel^®^ 2007.

### Ethical aspects

Access to information from the clinical histories was requested from the directors of hospitals in Montería, Planeta Rica, Sahagún, Tierralta, Lorica, and Cereté. The information provided was used for the benefit of the inhabitants in the region. The study always maintained the anonymity of the patients and adhered to ethical standards, scientific techniques, and administrative norms for health research from Colombia's Ministry of Social Protection, resolution Nº 008430 of 1993. The study was considered low risk and was approved by the ethics committee in the Institute of Tropical Biological Research at Universidad de Córdoba. 

## Results 

From analysis of the 1,965 cases (Data A), it was estimated that 76.2% were from the urban zone; the dengue: SD ratio was 7:1; 56.8% were men, 53.0% of the patients were younger than 14 years of age. The differences in the ratio dengue/SD by ages and social security healthcare scheme weren't significant. However, the differences urban-rural were significant (Table 1). Furthermore, differences urban-rural in the distribution of cases by social security healthcare scheme were observed. Twenty six, point two per cent of cases were in population no affiliated to health system, especially in rural localities (Table 2).

**Table 1.**
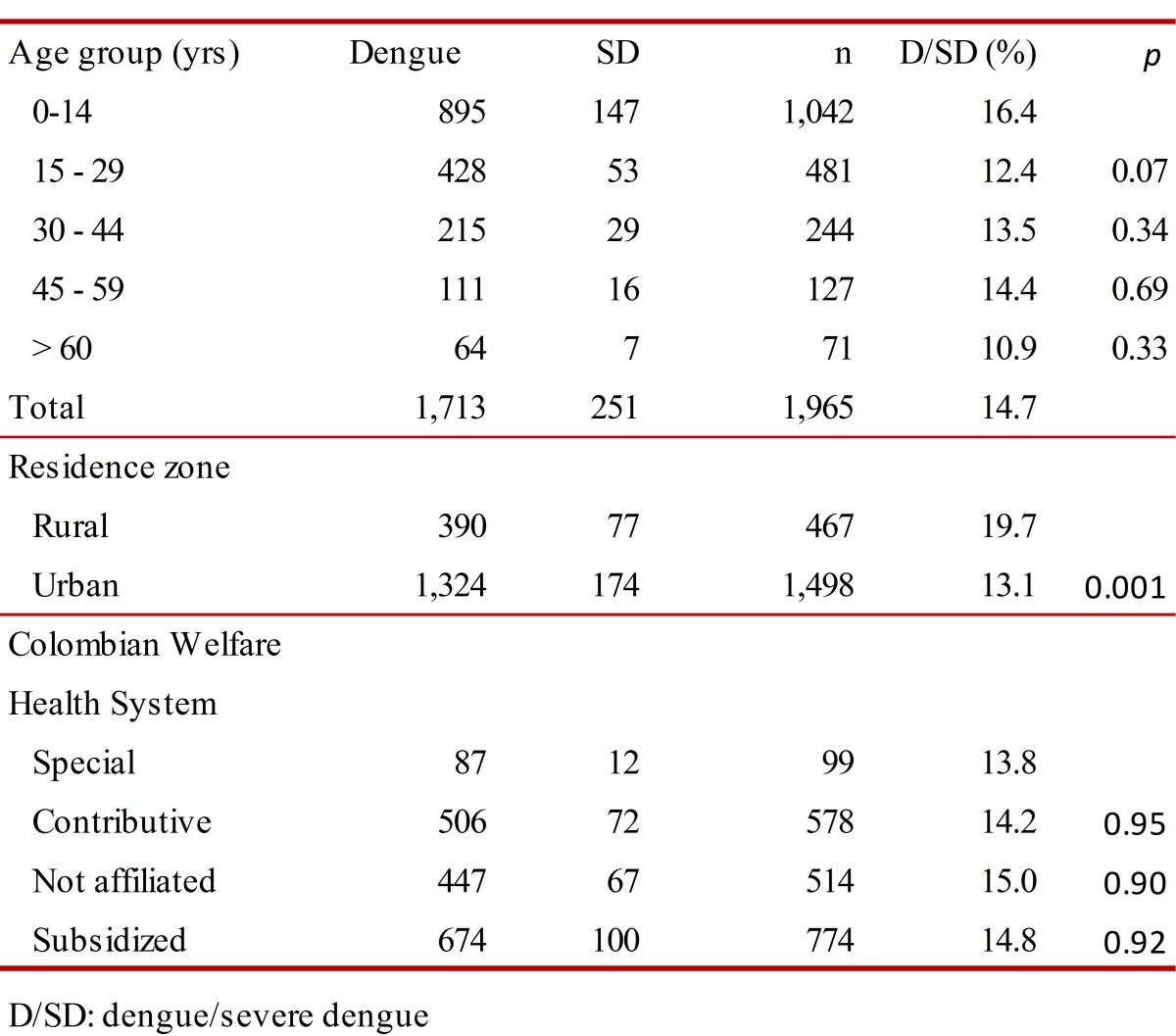
Characteristics of cases reported with diagnosis of dengue to the Health Direction of the Department of Córdoba. 2003-2010.

**Table 2.**
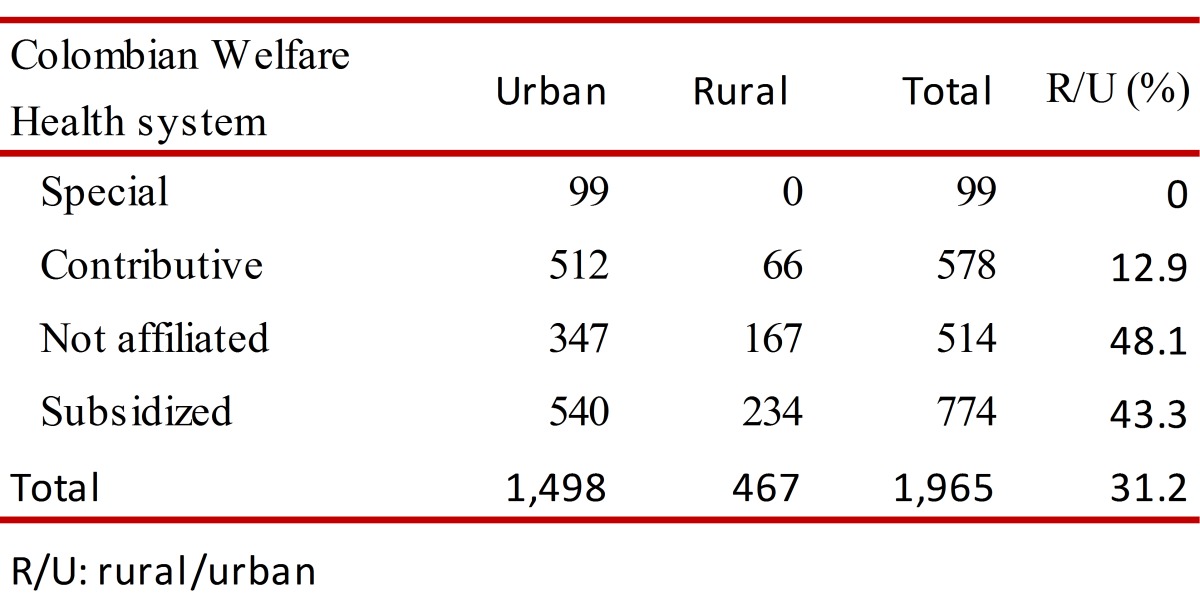
Distribution rural-urban and by health regime of cases reported with diagnosis of dengue to the Health Direction of the Department of Córdoba. 2003-2010.

**Figure 1.**
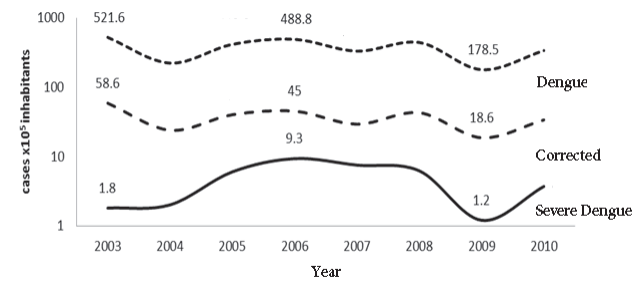
Incidence (cases x10^5^ inhabitants) of dengue in Córdoba. 2003-2010.

The mean incidence of dengue between 2003 and 2010 was 36.5 cases/10^5^ inhabitants (CI95%: 34.3-37.5) a minimum of 19.8 (2009) and a maximum of 60.4 (2003). With the adjustment for sub-reporting, the incidence could be between 178.5 and 521.6 cases/10^5^ inhabitants. The mean incidence of severe dengue was 4.7 cases/10^5^ inhabitants (CI95%: 4.3-5.0) (Fig. 1).

**Figure 2.**
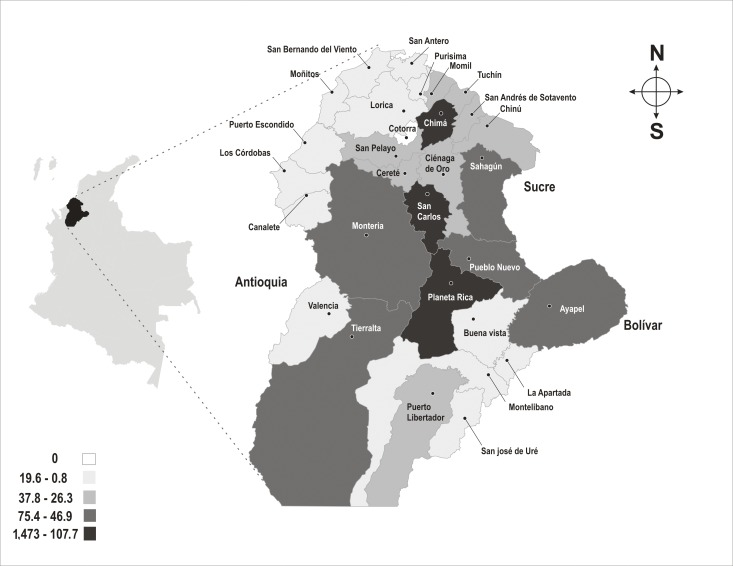
Incidence (cases x10^5^ inhabitants) of dengue per municipality in the department of Córdoba. 2003-2010.

The municipalities in the department of Córdoba with the highest incidence of cases of dengue between 2003 and 2010 were: San Carlos, Chima, and Planeta Rica with a mean incidence of 147.3, 146.7, and 107.7 cases/10^5 ^inhabitants, respectively (Fig. 2). 

### Mortality

The mean mortality rate due to dengue was estimate with database from the Direction of Health in the Department of Córdoba. In Córdoba between 2003 and 2010 was 0.3 cases/10^5^ inhabitants; the highest rate corresponds to with 0.6 x 10^5^ inhabitants, followed by 2007 and 2009 with 0.4 x 10^5^ inhabitants. Regarding the fatality rate, the department of Córdoba is below 1%, the highest fatality rate was in 2009 a rate of 1.9% was found, followed by the 2006-2007 period with a rate of 1.1%.

With respect to the findings in the 230 histories reviewed (Data B), laboratory results show that the mean total leukocyte count (neutrophils, lymphocytes, eosinophils, basophils, and monocytes) observed during the first test conducted on patients with dengue was 6,181 mm^3^ (CI95%: 5,973-6,389) and with SD it was 4,729 mm^3^ (CI95%: 4220-5238). The average platelet count observed during the first test conducted on patients with dengue was 118,793/mm^3^ (CI95%: 107,255-130,331) and in patients with SD 77,655 (CI95%: 59,640-95,670). Both differences were statistically significant (*p* <0.05).

The laboratory tests most often used (frequency of uses: test mean per patient) in patients with dengue and SD were platelet count (Dengue: 2,517; SD: 3,893), hemogram (dengue: 1.736; SD: 2.625), and hemoparasites (dengue: 0.977; SD: 1.054). Only IgM and IgG tests were performed for dengue in 21.3% of the individuals diagnosed with dengue (dengue: 0.98; SD: 0.339) (Table 3).

**Table 3.**
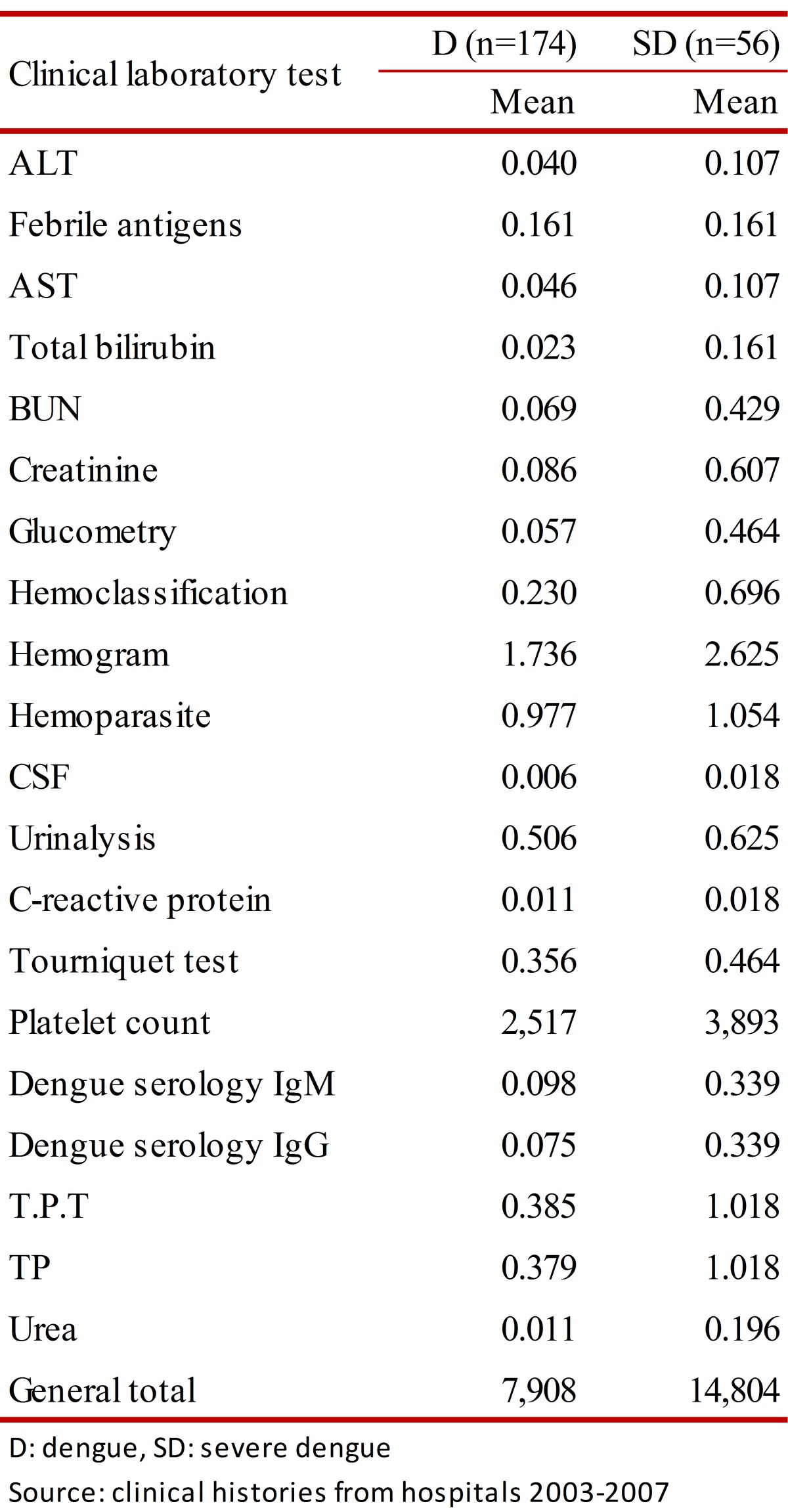
Mean of clinical laboratory tests from dengue patients, Cordoba. 2003-2007.

## Discussion 

This study summarizes any epidemiological aspects of dengue in the department of Córdoba, in the Colombian Caribbean, showing that the incidence of cases estimated from surveillance records from health authorities is not consistent with the behavior of the diseases transmitted by vectors that describe a significant increase, probably due to climate change and other causes 9,10. Correction for sub-reporting shows an incidence probably closer to reality, between 178.5 and 521.6 cases/10^5^ inhabitants. However, the non-adjusted results from the current study were similar to those from Venezuela in 2003, where incidence rate was estimated at 63.4 cases/10^5^ inhabitants 11; but do not coincide with those reported in Colombia; in Santander, where dengue incidence rates were between 113.4 and 268.7 cases/10^5^ inhabitants, respectively 12. These also do not coincide with international studies like Mexico and Brazil, where incidence was reported at 27.2 cases/10^5^ and 283.8 cases/10^5^, respectively 13,14, which shows the probable evidence of sub-reporting and other aspect as lack of diagnostic.

Cases reported of shock due to severe dengue produce mortality between 40 and 50% 15,16; but in this study, the mortality rate due to dengue was low. Literature has described that when medical attention is offered by professionals with knowledge of the dengue virus (physicians and nurses who know of its symptoms and know how to treat its effects) the fatality rate can be reduced to less than 1% 17. These results on the low mortality rate of dengue in Córdoba do not agree with Salgado *et al*. 18, who reported that Colombia was the country with the most cases of severe dengue and deaths due to this cause in America during 2004 and 2006. This could be because in Córdoba no systematic record is kept and the cases were undervalued or not recorded. 

Fatality results coincide with those from the Americas during 2001-2006, where a fatality rate of 1.2% was reported and with that from Mexico 19 in 2008 with fatality at 0.7%. This means that the vector control and management program from the Health Direction of the Department of Córdoba needs to improve strategies for vector control and comprehensive management of dengue, according to the entomological risk and the dynamic stratification of probable cases (epidemiological risk) with immediate and comprehensive actions.

According to place of residence, the number of patients from urban areas was higher (76.2%), which is similar to that found in Neiva (Colombia) (69.6%), Panama (86%), Brazil (70%), and Peru (90%) 14,20-22. In Colombia, approximately 70% of the population lives in urban zones. Growth of cities is associated to increased rural-urban migration due to multiple socioeconomic causes and forced displacement caused by armed groups, among others. From the epidemiological perspective, these aspects affect the rate of infectious contacts between mosquito vectors and humans and, hence, dengue epidemics and maintenance of the disease in the cities 23. Additionally, dengue is a disease mainly transmitted by the *A. aegypti *mosquito, which inhabits urban sectors 24,25, and the *A. albopictus *mosquito implicated as the second most important vector of dengue virus, unlike *A. aegypti*, *A. albopictus* proliferates in urban, rural, and jungle zones. Both mosquitoes evidence close cohabitation with humans and are of great importance at the social level 26. Other authors have reported that water tanks and containers to store water in the households were the breeding grounds during different most productive states of the mosquito 19,27. Cordoba has been a department with low coverage of drinking water and sanitation facilities in the past decades.

Regarding the most-often used laboratory tests, the results do not coincide with others that found a greater frequency of hemo-concentration associated to a strong plasma capillary leakage (presence of ascites) and upon diagnosis of severe dengue 28. Immunoglobulin M and IgG tests were only carried out for dengue in 21.3% of the individuals diagnosed with dengue (dengue: 0.98; SD: 0.339). This shows that perhaps hospitals do not timely diagnosis methods 29. 

The tourniquet test was carried out on 93 of the 230 patients, 84.9% (79/93) of the cases were positive. According to some authors 30-32, it is a useful tool to arrive at the diagnosis and it has also been documented as the main hemorrhagic manifestation in patients with dengue. Although other authors express that the lack of the tourniquet sign should not be considered 32, given that it can be negative in up to 60% of the cases, although after the state of shock it becomes positive.

Regarding socioeconomic conditions, only 39.4% of the patients were in the subsidized social security scheme and most came from low socioeconomic levels in impoverished sectors with deficiency of healthcare services. Villar 33 and Kouri *et al*. 34, described that deterioration of healthcare systems produces substantial increases in the incidence of cases of dengue and gradually increases severe dengue. Although some authors consider that poverty is not related to the disease, however, it has been determined that the magnitude of the risk depends on the materials with which the dwelling is constructed, its sanitation conditions, and its surroundings 35.

## Conclusions 

Study permitted establishing the dengue situation in an endemic zone with high sub-reporting; we managed to observe deficiencies in vector management and that etiological diagnosis is only made in low proportions. Furthermore, the low capability for diagnosis and treatments in hospitals show problems of access and non-quality health services. 
